# Early days of the pandemic—The association of economic and socio-political country characteristics with the development of the COVID-19 death toll

**DOI:** 10.1371/journal.pone.0256736

**Published:** 2021-08-31

**Authors:** Manuel Holz, Jochen Mayerl

**Affiliations:** Institute for Sociology, Faculty of Behavioural and Social Sciences, Chemnitz University of Technology, Chemnitz, Germany; University of Western Australia, AUSTRALIA

## Abstract

This article examines cross-national differences in growth of deaths by COVID-19 over time in the first phase of the pandemic, during the time period of 31^st^ December 2019 to 2^nd^ April 2020. We seek to understand and explain country level reaction in the initial period of the pandemic. We explore socio-economic and socio-political country characteristics as determinants of deaths per day and we examine whether country characteristics act as moderating factors for different growth patterns of deaths per day over time. The country characteristics include variables about economy, globalization, health care and demography. We examine data published by the European Center of Disease Prevention and Control (ECDC) in combination with World Bank data and a webscraping approach. Using a conditional growth model specified as a multilevel regression model with deaths by COVID-19 per day as the outcome variable, we show that economic variables are not significantly associated with decrease or increase of deaths by COVID-19. In contrast, variables about national health care mitigate the impact of the pandemic. Demography shows expected effects with an increase of growth of deaths in countries with a higher percentage of people older than 65 years. Globalization predicts the death toll as well: Social interaction between people is deadly on a short-term scale (in the form of tourism). Our results mirror frequent demands for global investment in national health systems.

## 1. Introduction

The recent pandemic of Corona Virus (COVID-19) comes with extraordinary challenges for all countries and residents all over the world. We want to contribute to the question of which socio-economic and socio-political characteristics of countries are associated with deaths per day caused by COVID-19 at the very beginning of the pandemic. Crucial to our question is how infection activity, in the form of deaths, behaves in the initial period of the pandemic with respect to quantifiable metrics of a country. This analysis offers a general image on pandemic performance and preparedness when no specific measures to mitigate the spread of the virus have taken place yet.

To be more precise, we look at effects of economic factors (national wealth and economic growth), global contacts (e.g. national inbound tourism), health factors (e.g. national health expenditures and health infrastructure), and socio-demographic factors (e.g. age structure).

The paper follows an explorative approach guided by two research questions: *Firstly*, analyzing factors affecting growth over time: Which country characteristics are associated with an increase or decrease of deaths per day caused by COVID-19 in the initial wave? *Secondly*, do country characteristics moderate the positive or negative growth of deaths per day? The last question is essential for the analysis of country differences during the pandemic, since this means we are asking whether economic or socio-political country characteristics play a crucial role in the increase or decrease of deaths per day. Our main contribution to the existing literature lies in the ability to estimate the “velocity” of the development of the death toll dependent on country level characteristics.

Subsequently, we run a longitudinal multilevel analysis of macro variables of 61 countries. We examine data published by the European Center of Disease Prevention and Control (ECDC) in combination with World Bank data and a webscraping approach.

## 2. Analytical background

We propose that the spread of infectious diseases due to country level factors can be summarized in four pathways: via economic competition, degrees of connectivity, population dynamic and protective measures.

Economic competition forces livestock manufacturers and farmers to create conditions under which diseases can spread more easily. Minimizing costs in production to be able to compete in globalized markets often means keeping a higher density of animals in the same area and reducing the quality of pet food and care. It was shown that the animal to human transmission of the Nipah virus was largely due to the change in farming practices caused by economic expansion in combination with habitat destruction [1]. Bilal et al. discuss the association of periods of macroeconomic growth with increases in population mortality and show that social protection policies can mitigate negative effects of economic growth on health [2]. Toffolutti and Suhrcke address the connection between austerity (i.e. tight fiscal policies) and mortality, but they also show that fiscal stimuli tend to increase death rates [3]. From perspective of the welfare state, higher national and individual health is associated with a better resource allocation, better standard of living and better medical measures. Overall, the connection between population health and economic factors seems to be complex with conflicting effects.

Globalization and population health (health status and health outcomes of groups and individuals) are connected through interrelated patterns driven by demographic and social changes, environmental impacts and economic activities, pointing to negative as well as positive associations between globalization and health [4]. With regards to global disease epidemics, the recent WHO report on “A world at risk” [5] clearly addresses the connection between an increasingly globalized world and health emergencies. Higher degrees of global connectivity enable a faster spread of diseases. Without human-made paths, disease spread fizzles out more quickly. According to Baker, higher degrees of transportation and cross-border interaction in the form of travellers (tourism), trade and traffic increase the degree of connectivity between potential disease nodes [6]. Globalization can therefore be seen as a catalyst for disease diffusion [7].

Since economic expansion is linked to population ageing [8], susceptibility to diseases and infections increases along with economic expansion. A side effect of higher educational attainment, labour market participation and the subsequent decline of fertility rates is the increase in average population age and therefore a higher prevalence of pre-existing diseases and lower levels in physical functioning (Baker 2015, Ceddia et al. 2013, Stenholm et al. 2015) [6, 7, 9].

In order to cope with exposure to potential health hazards, individuals will have to use resources provided by their environment [10]. The equipment of and access to the respective health care system is therefore predictive in both the spread of disease and its fatal consequences. Resource gaps and overload of medical institutions have been shown to be a primary driver in the development of deaths during pandemics [11].

Recent studies considered similar indicators and their effect on the death toll by COVID-19 in cross-country analysis. In one study [12], the amount of annual tourists was a strong predictor for cumulated deaths and cases by COVID-19 infection, as well as hospital beds and number of nurses per 1000 people. In Farzanegan et al. a more global measure of globalization showed predictive power on the number of reported COVID-19 cases, but not on confirmed deaths [13]. The cited studies offer elemental insight using both cross-sectional and cumulated data in a basic linear regression framework without addressing the hierarchical and longitudinal nature of the data. We complement the existing literature by offering a longitudinal, multi-level approach, which permits us to estimate cross-level interactions on the daily death toll. Further, we offer a measure of country specific COVID-19 testing capacity, which gives additional robustness to our results.

Other pandemic drivers such as the institutionalized population [14] or the reliance on industries which employ no social distancing measures (e.g. working from home) are not directly included in our analysis. However, these drivers are linked to a variety of variables in our model. For example, country level human capital (captured by high school enrolment rates and GDP) is a determining factor for a shift towards more employment in the service and communication industries [15], which facilitates social/physical distancing measures. Further, hospital beds have been shown to be inversely correlated to incarceration rates [16, 17]. In order to maximize the sample size, we rely on the more global indicators, such as GDP, educational enrolment rates and number of hospital beds.

In the following analyses, we address the question of whether national economy, health system, global connectivity and demographic characteristics are associated with deaths by COVID-19 in a multivariate setting. We place special focus on whether such country characteristics moderate the growth of deaths per day.

**Fig 1:**
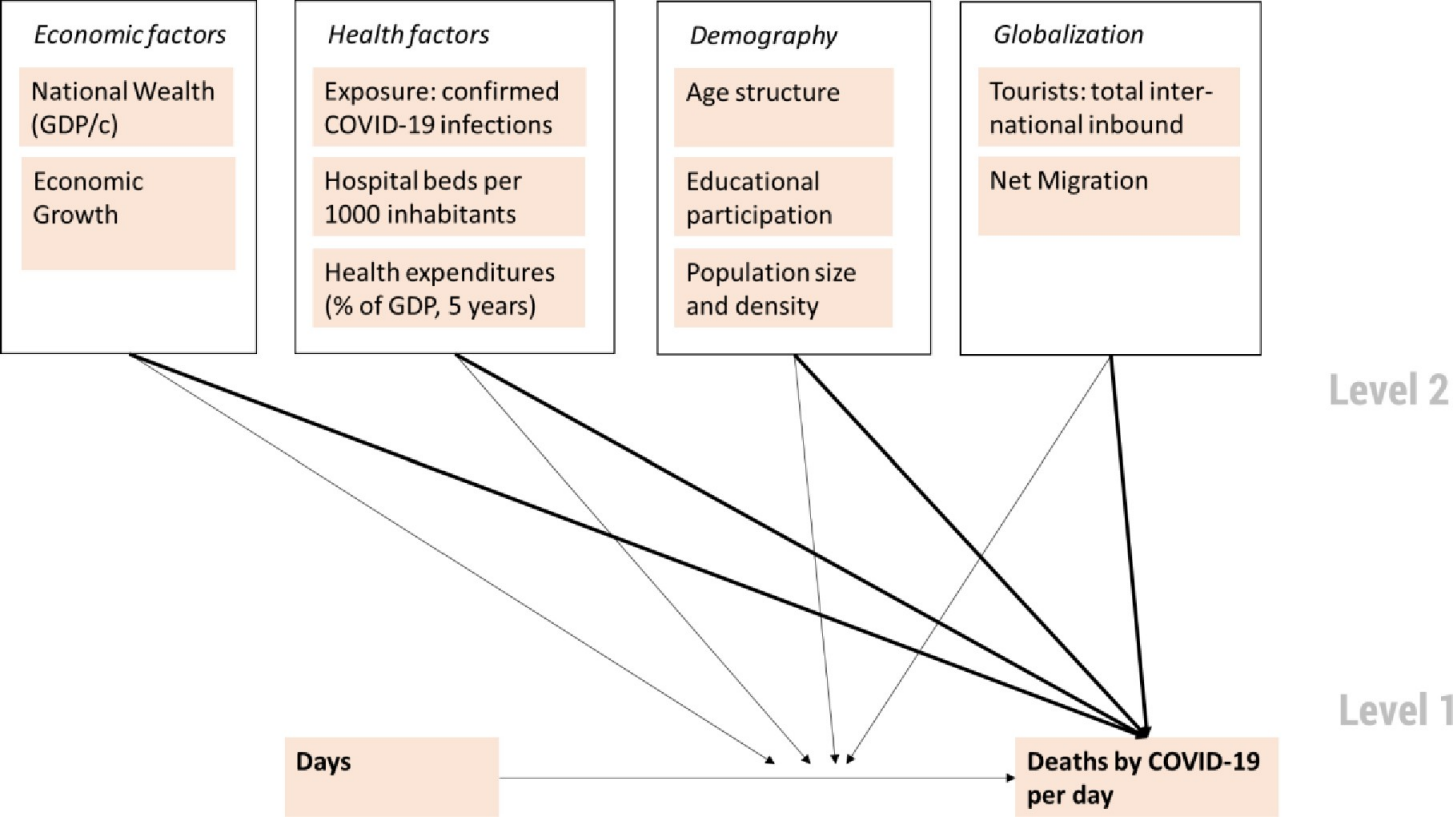
Analytical multi-level model.

Fig 1 shows our multilevel model of direct effects as well as moderator effects of economic factors, health factors, demography and globalization (Level 2) on the amount and growth of deaths per day over time (Level 1).

## 3. Data and methods

We are interested in infection activity at the beginning of the pandemic, when various counter-measures and long-term strategies had not yet been fully implemented. This raises the question of what to consider the “initial period”, especially considering lockdown measures (restrictions of individual and mass movement to achieve spatial distance) vary in strength and time of first implementation. E.g. Italy started the first nation-wide lockdown on March 03, 2020, while Germany started on March 23, 2020. The United Kingdom did not have a nation-wide lockdown at all. We use the average duration between infection and death to determine where to set the cut-off point. According to the literature, this is 16–18 days [18, 19]. Using this approach, we try to gather data on the death toll, even if first lockdown measures have already taken place.

About 50% of the countries studied in our dataset are from Europe. We used a cut-off point of the lower bound 16 days from the day on where the majority of European countries were already at their first lockdown (March 17), which gives us April 02, 2020. Therefore, our results are restricted to a relatively short time period of approximately three months (2019/12/31–2020/04/02, total 94 days)–which we consider the beginning of the pandemic (except China which is not included in our analysis).

For the analysis, we matched data provided by the European Center of Disease Prevention and Control [20], World Bank [21] and international health ministries. ECDC data contained country-level daily numbers for reported cases of COVID-19 infections and deaths. World Bank indicators provided macroeconomic information by country. Finally, we applied a webscraping approach to gain a database with a collection of health ministry press releases to obtain an approximation for country level COVID-19 testing. The health ministry press releases are collected on the Wikipedia page for Covid-Testing (see https://en.wikipedia.org/wiki/COVID-19_testing). Webscraping press releases or metadata of public institutions to gain lacking data on public health issues has been applied to link incarceration and HIV rates [22] and to detect community autism spectrum disorder rates [23]. Further, Wikipedia has been deemed helpful for public health needs [24] and especially for modelling trends of the Coronavirus outbreak (Doğaner, 2020) [25]. The webscraping process is carried out as follows: making use of the editing page history of the Wikipedia Covid-Testing website and the included metadata (date and time), all tables of the press releases with their respective number of applied national Covid tests were gathered for the defined time period. Naturally, there exist several versions of a table per day, since the tables are updated multiple times a day. We chose the last update of a table in a given day to define the testing capacity of a country on a given day. Website content is retrieved either by using a browser plug-in to locate relevant HTML objects or manually via the browser’s developer mode when necessary.

Our data consists of two levels: daily reports on deaths by COVID-19 (Level 1) nested within each country (Level 2) (see Fig 1). To deal with this type of hierarchical data, we estimate a multilevel regression with random intercepts and random slopes for longitudinal data [26, 27]. Multilevel models have several advantages when dealing with hierarchical data, including corrected standard errors and the ability to estimate cross-level interactions. This allows us to investigate the influence of country level context variables on the daily reported death toll and the difference of the slopes of the change over time. To detect macro level effects on the course of the development of daily deaths, interactions of growth over time with macro variables are calculated.

After applying listwise deletion (dropping countries form the dataset in case of missing values in country level data), the final data set for multilevel analysis includes 61 countries from Asia, Europe, North and South America, Middle East, and Oceania. The country codes of included countries are: ARE, ARG, ARM, AUS, AUT, BEL, BHR, BLR, BOL, BRA, CAN, CHE, CHL, COL, CRI, CZE, DEU, DNK, ECU, ESP, EST, FIN, FRA, GBR, GRD, HRV, HUN, AND, IND, IRL, IRN, ISL, ISR, ITA, KAZ, KOR, LTU, LVA, MEX, MLT, MYS, NOR, NPL, NZL, PAN, PER, PHL, POL, PRT, ROU, RUS, SGP, SRB, SVK, SVN, SWE, THA, TUR, UKR, URY, and USA. According to Hox (2010: 235) it is suggested having a sample size of at least 50 units on level 2 and at least 20 units on level 1 to analyze cross-level interactions, which is the focus of this paper. Following to this rule of thumb, with 61 countries (level 2) and 94 days on level 1, the sample size in our study is sufficient on both levels.

## 4. Measures

**Fig 2:**
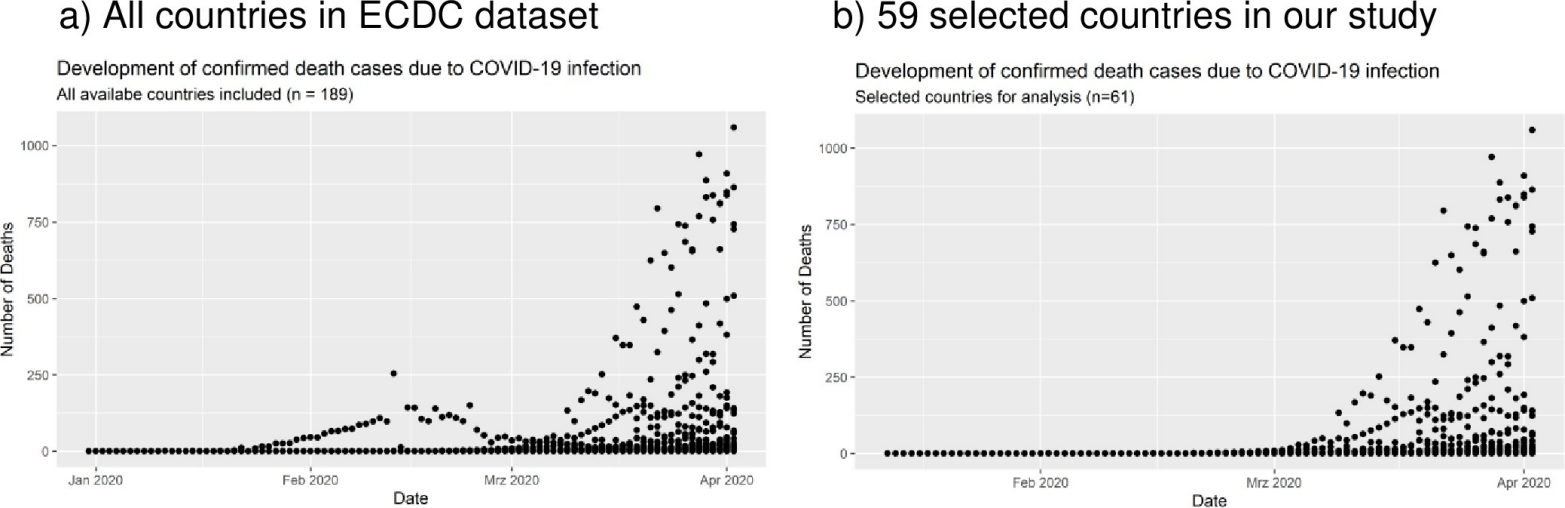
Deaths by COVID-19 over time (31^st^ December 2019 to 2^nd^ April 2020). a) All countries in ECDC dataset. b) 61 selected countries in our study.

On Level 1, the dependent variable is measured as the reported total amount of deaths per day due to COVID-19 infection for each country between 2019/12/31 and 2020/04/02 (94 days). Further, we take days before the last measurement occasion (2020/04/02) in each country as the independent time variable. The variable is 0-centered, where 0 corresponds with the last day of reported cases (the days before the last day are therefore represented with negative numbers). This allows us to take a look at the effects of country-level characteristics on the last day of measurement occasions. Since deaths per day over time do not occur in a linear function (see Fig 2B), we took the logarithm of the day counter to estimate a positive logarithmic function (because of the negative numbers in „days before last measurement occasion“, we took the LOG in the following way: days(log) = (-1) * log((days before last measurement occasion*-1)+1). The result is a scale which still is at “0”at the last time point of our measurement period.

### 4.1 Economic factors

Variables included are GDP per capita based on 2011 purchasing power parity of international Dollars (divided by 10.000) and the five-year-averaged GDP growth rate. GDP has shown to be a valuable predictor for health outcomes [28, 29].

### 4.2 Demographic factors

Variables include population age (percentage of 15 to 64 year olds, percentage of 65 and above year olds), enrolment rate to secondary education (percentage of total enrolment to corresponding age group), total number of inhabitants (divided by 100.000) and population density (divided by 1000).

### 4.3 Globalization factors

We include net migration (total number of migrants less the annual number of emigrants divided by 100.000) and annual total international inbound tourists (divided by 100.000) in line with previous studies on globalization and the COVID-19 outbreak [12, 13].

### 4.4 Health factors

Variables include confirmed COVID-19 infections (country level averaged divided by 1000) to get a measure of national exposure, hospital beds per 1000 people and five-year-averaged health expenditures as percentage of the GDP. These measures are common in social science and health research [28]. The amount of conducted COVID-19 infection tests was reported for each country in different intervals. It is essential to control for this measure since testing is assumed to be endogenous. Some countries only reported for one day or the total applied tests. For each country the number of conducted tests was averaged across all available time points (and divided by 1000) to get national COVID-19 testing as a control variable in our statistical analysis.

In order to get interpretable zero values of all level 2 variables, the overall or grand mean of each variable was subtracted from the values of the respective variable [26 pp61]. This grand mean centering results in receiving a zero value that corresponds to the mean of the variable over all countries.

## 5. Results

The appropriateness of conducting a multi-level analysis is given by calculation of the Intraclass correlation (ICC) of the unconditional intercept-only model. The ICC reflects the share of variance that can be traced back to the group (country) level and is suggested to be larger than 0.10 [26 pp244]. Our results reveal that the proportion of variance of deaths per day is 15.4% at the country level. The predictor model (including level 2 covariates) compared to the unconditional growth model (without level 2 covariates) shows a significant improvement in the Chi^2^ deviation (Chi^2^ = 220.8, df = 27, p = 0.000), justifying our multi-level model.

Table 1 shows the results of the multilevel regression. Due to the fact that all country variables were mean centered, all reported effects can be interpreted in a way that all other variables being on their mean value. We used nested models where we successively added cross-level interactions from the theoretical blocks as outlined in Section 4, while controlling all models for sociodemographic background and confirmed COVID-19 infections.

**Table 1:** Worldwide deaths by COVID-19: Multilevel analysis of longitudinal country-level data (31^st^ December 2019 to 2^nd^ April 2020).

			Estimates (SE)		
Model	1	2	3	4	5
(Intercept)	113.86*** (11.78)	113.87*** (11.77)	113.91*** (11.70)	113.94*** (11.48)	113.94*** (11.36)
**Level I predictors:**					
Days(log)	68.38*** (10.24)	67.21*** (10.55)	68.64*** (9.43)	69.52*** (7.58)	69.30*** (7.11)
**Level II Country characteristics:**					
Economic factors:					
National Wealth (GDP/c, 10.000ppp)	-13.99 (10.00)	-14.74 (10.01)	-13.89 (9.93)	-13.49 (9.73)	-13.92 (9.66)
Economic Growth (%, 5 years)	-10.83 (7.71)	-10.76 (7.71)	-10.71 (7.65)	-10.60 (7.50)	-11.50 (7.44)
Health factors:					
Exposure: confirmed COVID-19 infections	359.52*** (59.83)	360.21*** (59.80)	360.91*** (59.42)	362.24*** (58.27)	363.22*** (57.71)
Hospital beds per 1000 people	-18.86** (6.67)	-18.82** (6.67)	-19.30** (6.63)	-18.39** (6.50)	-19.22*** (6.44)
Health expenditures (% of GDP, 5 years)	-905.12 (834.25)	-901.66 (833.78)	-995.20 (829.03)	-900.28 (811.92)	-969.79 (805.64)
Average COVID-19 tests per day	**25.88**^**+**^ (13.04)	**25.82**^**+**^ (13.02)	**25.79**^**+**^ (12.95)	**25.39**^**+**^ (12.70)	25.28* (12.57)
Demography:					
Age: % of 65+ year olds	872.87* (412.87)	868.77* (429.55)	903.96* (410.16)	845.25* (402.04)	898.34* (398.34)
Age: % of 15–64 year olds	-103.18 (430.96)	-88.50 (430.81)	-102.24 (428.0)	-116.55 (419.72)	-99.78 (415.89)
Enrolment rate to secondary education	65.94 (86.94)	69.14 (86.92)	66.34 (86.35)	67.94 (84.66)	65.58 (83.89)
Population size	-0.29*** (0.08)	-0.29*** (0.08)	-0.29*** (0.08)	-0.29*** (0.08)	-0.29*** (0.08)
Population density	10.57 (14.70)	11.08 (14.69)	10.13 (14.59)	10.28 (14.31)	10.60 (14.18)
Globalization:					
Tourists: total international inbound	2.85*** (0.96)	2.85*** (0.96)	2.84*** (0.95)	2.94*** (0.94)	2.92*** (0.93)
Net Migration (n_migrants_-n_emigrants_)	-12.69*** (2.29)	-12.67*** (2.29)	-12.61*** (2.28)	-12.97*** (2.23)	-12.91*** (2.21)
**Cross-Level-Interactions:**					
Days(log) * National Wealth	-	-13.38 (7.57)			-4.82 (5.83)
Days(log) * Economic Growth	-	0.73 (5.66)		-	-8.82 (4.63)
Days(log) * Hospital beds	-	-	-10.40* (5.01)	-	-9.66* (3.92)
Days(log) * Health expenditures	-	-	-1589.62*** (532.20)	-	-791.85 (497.46)
Days(log) * Tourists	-	-		2.11*** (0.58)	1.83*** (0.58)
Days(log) * Net migration	-	-		-7.28*** (1.30)	-6.73*** (1.30)
Days(log) * Exposure confirmed infected	219.88*** (20.35)	233.11*** (21.94)	245.90*** (22.20)	248.90*** (28.26)	257.89*** (28.90)
Days(log) * Age 65+	164.03 (228.91)	308.45 (240.43)	803.03** (302.95)	7.31 (171.45)	589.24* (251.18)
Days(log) * Age 15–64	-74.88 (322.07)	143.11 (345.08)	8.47 (311.80)	-146.14 (240.89)	15.08 (259.40)
Days(log) * Education rate	40.29 (64.51)	94.48 (72.41)	66.34 (86.35)	61.26 (47.63)	39.87 (53.18)
Days(log) * Population Size	-0.01 (0.06)	-0.02 (0.06)	-0.03 (0.06)	-0.15*** (0.05)	-0.14*** (0.05)
Days(log) * Population Density	3.20 (10.41)	12.66 (11.58)	-4.07 (9.85)	2.54 (7.59)	1.80 (8.56)
**Random Effects**					
σ^2^_eij_	1330	1330	1330	1331	1332
σ^2^_u0j_ _intercept_	7278	7270	7173	6889	6746
σ^2^_u1j_ _slope of days(log)_	5721	5582	4814	2972	2576
**Fit Measures**					
AIC	24998	24988	24972	24964	24933
BIC	25137	25139	25123	25115	25107
LogLik	-12475	-12468	-12460	-12456	-12436
Observations	2453	2453	2453	2453	2453
n _countries_	61	61	61	61	61

Remarks

Sig codes: p ≤ 0.001***, p ≤ 0.01 **, p ≤ 0.05 *, p ≤ 0.06^**+**^.

days(log): -LOG of days before last measurement occasion; Restricted Maximum Likelihood (REML) estimator; all country characteristics were grand mean centered; Fit measures with Maximum Likelihood (ML) estimator; Intra Class Coefficient (ICC) of the unconditional model: 15,4%; software: lme4-package in R.

*Firstly*, as expected, increasing days since infection is significantly associated (model 1: p = 0.000, model 5: p = 0.000) with increasing daily reported deaths (positive logarithmic function). As the data stem from time points during the COVID-19 pandemic period, the results are in line with expectations about a strong positive logarithmic growth over time, where the slope does not decrease over time (as we would expect when a pandemic comes to its end).

*Secondly*, the effects of Level 2 country characteristics are the effects for day “0”, which is the last day of our data occasions (2^nd^ April 2020). Therefore, these effects can be treated as a cross-sectional study. The level 2 variables explain deaths on the last day of observation in the following way: the economic predictors don’t show any significant effect on deaths. Neither GDP per capita (model 1: p = 0.168, model 5: p = 0.156), nor annual GDP growth (model1: p = 0.167, model 5: p = 0.129) reach statistical significance.

Health factors show significant negative effects of hospital beds per 1000 people on the number of deaths. This effect is stable over all models 1–5 (model 1: p = 0.007, model 5: p = 0.005). On the other hand, health expenditures show no significant effect on the number of deaths in all models (model 1: p = 0.238, model 5: p = 0.235). The average COVID-19 testing capacity has a weak significant effect on the death toll (model 1: p = 0.053, model 5: p = 0.050).

Regarding the socio-demography of a country, it can be shown that the amount of people above the age of 65 is significantly associated with a higher number of deaths (model 1: p = 0.040, model 5: p = 0.029). This result is in line with frequent reports that identify people of older age belonging to risk groups of COVID-19 fatalities. The coefficient for educational enrolment shows no significance on a statistically relevant level (model 1: p = 0.452, model 5: p = 0.438). Both effects of globalization indicators are statistically significant. The amount of migrant influx (model 1: p = 0.000, model 5: p = 0.000) to a country decreases the death toll on the last day of observation, whereas the amount of incoming tourists increases (model 1: p = 0.005, model 5: p = 0.003) the death toll.

*Thirdly*, now coming to the most interesting longitudinal part, we observe a significant variation of the growth factor (i.e. slope of days) between countries. In other words: the growth of COVID-19 deaths over time is not the same across the countries. To explain these variations in growth, we estimate cross-level interactions between country characteristics (Level 2) and days (Level 1). Looking at these interaction effects, the following observations can be made:

In the realm of demography, population age (age > 65), does not determine deaths per day, when considered individually (model 1: p = 0.477), but a positive significant effect appears when controlling for health system indicators (models 3 and 5, p<0.05). Since population age correlates positively with hospital beds and death toll, and hospital beds correlate negatively with death toll, the direct effect of age is neutralized, when excluding health system indicators (model 1, 2, and 4). Enrolment to secondary education does not show statistically significant effects on the death toll per day in any given model (model 1: p = 0.535, model 5: p = 0.457).

The effect of population size is only significant in models 4 and 5 (p<0.01) when globalization indicators are included. In these models, countries with higher values in population size experience a decrease in the number of deaths per day. On the other hand, in models with no explicit control for tourism and net migration (models 1, 2, and 3), the effect of population size on death toll is not significant (p>0.05). This can be explained by contrary correlations of population size with tourism (positive correlation) and net migration (negative correlation), leading to a reduction of the significance of the effect of population size in the uncontrolled models. Population density has no significant effect on daily deaths across all models (model 1: p = 0.760, model 5: p = 0.834).

Further, no economic indicators can explain the differences between countries regarding growth of daily deaths over time, i.e. countries with varying levels of economic wealth (model 2: p = 0.083, model 5: p = 0.412) or growth (model 2: p = 0.897, model 5: p = 0.062) do not differ in increase or decrease of deaths per day over time.

Without control for further factors (model 3), both variables concerning the health system have a statistically relevant effect on daily deaths: countries with more hospital beds per 1000 people have lower slopes in the outcome variable than countries with less hospital beds (p = 0.043). In other words, health infrastructure like hospital beds decreases the growth of deaths per day. Similarly, government health spending decreases the growth of deaths over time (p = 0.004). Controlling for further factors (model 5), however, leads to a loss in statistical significance in health expenditures (p = 0.118). The cross-level-interaction for hospital beds per 1000 people stays substantial and statistically significant (p = 0.017).

Looking at factors of globalization, the influx of tourists has a significant effect on the course of the development of reported death cases: countries with more annual tourists show a faster growth in COVID-19 deaths than countries with less annual tourists (p = 0.001). The effect is weakened, but still significant when further variables are controlled for (p = 0.003). In model 4 (p = 0.000), as well as in the final model (p = 0.000), net migration has a significant effect on the growth of the death rate. Countries with higher levels in net migration have a slower growth of the COVID-19 death rate.

As a robustness check, we re-estimated all models including country fixed effects (see Table 2 in the S1 Appendix). Here, all models include country dummies in order to address unobserved heterogeneity between countries. This results in a country fixed effects model in which all country differences are controlled for on level 2 which allows an unbiased identification of the cross-level interactions. Overall, regarding effect size and significance, results remain stable and comparable. Hence, the identified cross-level interactions are not biased by unobserved country differences.

**Fig 3:**
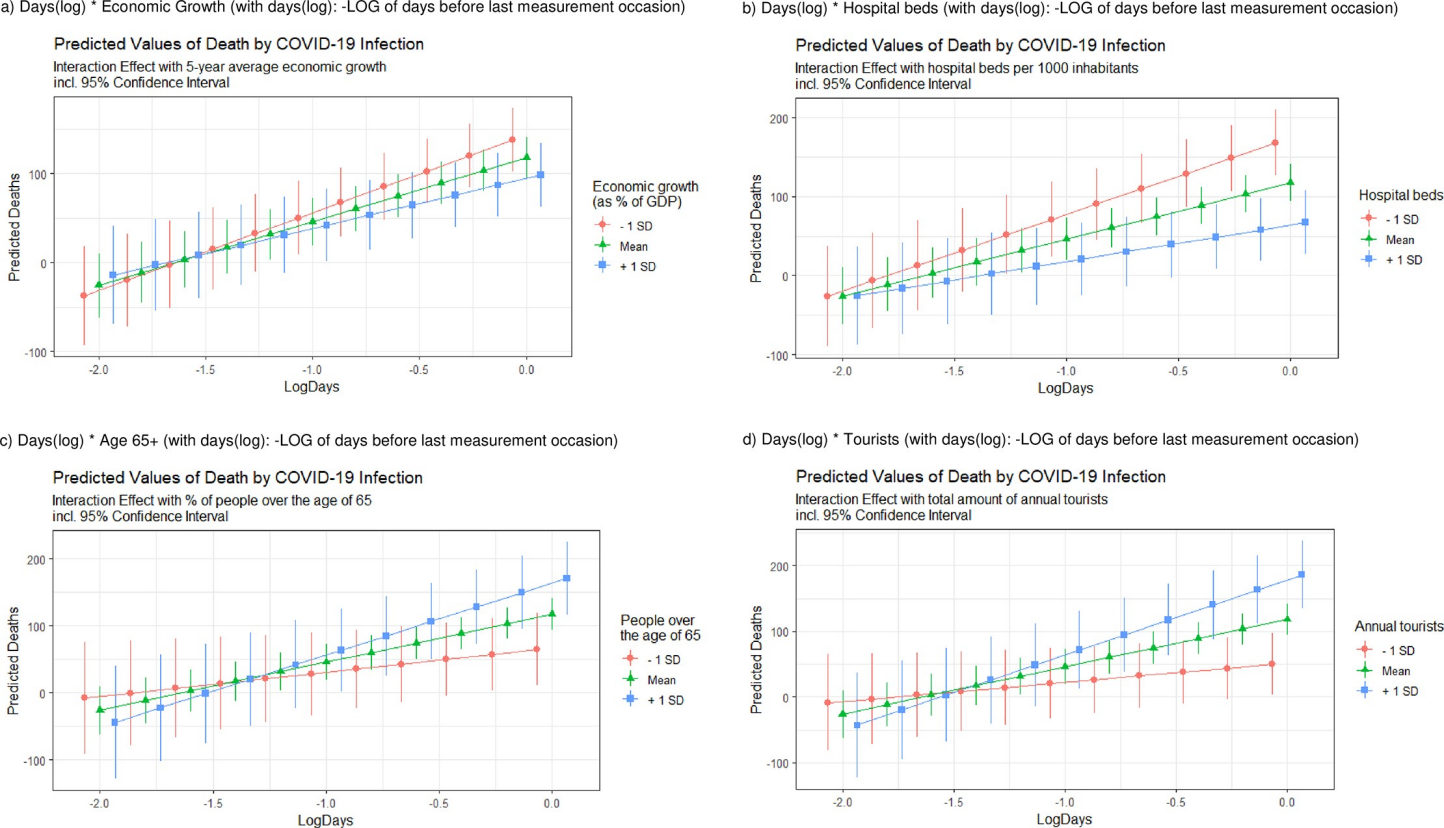
Visualization of four moderator effects (line positions were dodged for better visibility and to avoid over plotting). All coefficients represent the value for the exact same respective log-day). a) Days(log) * Economic Growth (with days(log): -LOG of days before last measurement occasion). b) Days(log) * Hospital beds (with days(log): -LOG of days before last measurement occasion). c) Days(log) * Age 65+ (with days(log): -LOG of days before last measurement occasion). d) Days(log) * Tourists (with days(log): -LOG of days before last measurement occasion).

Fig 3 shows a visualization of four selected cross-level interactions (economic growth, hospital beds, age 65+ and tourism).

## 6. Discussion and conclusion

Our results reflect what health and pandemic scholars [5] already emphasized frequently in the past as well as recent results on the issue [12, 13]: the course of the fatal effects of global disease outbreaks is in the hands of national commitment and investment in favour of a strong health care system. Access to health care can therefore mitigate the first impact of a pandemic. In both the cross-sectional and longitudinal case, hospital beds per 1000 people showed a buffering capacity on the death toll. More resources, which make health care more accessible to a broader part of the population, can translate into less virus fatalities. However, this health care must manifest itself in services accessible more directly to the population in order to reduce pandemic deaths, since health expenditures does not show significant effects in the final model.

The economy alone cannot save lives. As our results have shown, economic predictors do not explain the development of the death toll. The literature made clear [2, 3] that without implications for the social welfare state and public services, economic growth itself does not necessarily translate into the wellbeing of the citizens.

Globalization has predictive power concerning the death toll, in line with Farzanegan et al. [12] and Farzanegan et al. [13]. The data confirmed the identified threat of globalization on the accelerated emergence of new infectious diseases and its detrimental effects [4]. The influx of people to a country increases the death toll in the short term (in the form of tourism) and decreases the death toll in the long-term (in the form of migration). The spread of infectious diseases by travel enables infectious individuals to move in short time intervals across multiple national borders [6]. The negative effect of the net migration variable may point to a “Healthy migrant effect” [30, 31] on an aggregate level. This effect describes that those migrants who arrive in the host country typically have higher average health levels than the host population, which, inter alia, is attributed to health selection effects. Given that all other factors are equal (e.g. population size), the influx of migrants, which are relatively healthier, leads to a higher share of healthy individuals in a population with lower rates of pre-existing conditions associated with higher death tolls. Further research is needed to test more adequately the mechanisms behind this finding. Demography shows some expected effects, as growth of deaths is higher in countries with a higher percentage of people older than 65 years.

Despite our efforts to identify country characteristics which are linked to deaths by COVID-19 per day, potential shortcomings must be addressed. First, the data quality of reported infections and deaths relies on the governments’ public relations as well as on country-specific limitations to test for COVID-19 infections. Second, as the country selection is not randomly drawn and missing values occurred due to the webscraping process, there might be a selection bias in our data that could limit the generalizability of our results (as it is the case in most cross-national studies).

In the public policy context of making decisions under a restricted amount of time, where mobilization and efficient channelling of resources is crucial to maximize the reduction of the death toll in times of a pandemic, our results have the following implications. Since factors such as population age, economic wealth and population size are nearly impossible to stimulate, to manipulate or to vary within few days or weeks, factors concerning globalization in the form of border interaction are more elastic. Decreasing tourism activity by restricting or shutting down national borders, as it was part of many lockdowns in the first wave, has the potential to mitigate the spread of the virus and the load on hospitals and the health system. In a medium-term perspective, our results mirror frequent demands for global investment in national health systems. Meeting goals in core capacities of public health, in the form of a well-equipped national health care system, e.g. hospital beds, is the “first line of defense” in dealing with short term health crises [32].

## Supporting information

S1 Appendix (DOCX)

## References

[1] Epstein JH, Field HE, Luby S, Pulliam JRC, Daszak P. Nipah virus: impact, origins, and causes of emergence. Curr Infect Dis Rep 2006; 8(1):59–65. doi: 10.1007/s11908-006-0036-2 16448602 PMC7088631

[2] Bilal U, Cooper R, Abreu F, Nau C, Franco M, Glass TA. Economic growth and mortality: do social protection policies matter? Int J Epidemiol 2017; 46(4):1147–56. doi: 10.1093/ije/dyx016 28338775

[3] Toffolutti V, Suhrcke M. Does austerity really kill? Econ Hum Biol 2019; 33:211–23. doi: 10.1016/j.ehb.2019.03.002 31003198

[4] McMichael AJ. Globalization, climate change, and human health. N Engl J Med 2013; 368(14):1335–43. doi: 10.1056/NEJMra1109341 23550671

[5] Global Preparedness Monitoring Board. A world at risk: annual report on global preparedness for health emergencies. Geneva: World Health Organization; 2019. Available from: URL: https://apps.who.int/gpmb/assets/annual_report/gpmb_annualreport_2019.pdf?utm_source=ottawamatters.com&utm_campaign=ottawamatters.com&utm_medium=referral.

[6] Baker DMA. Tourism and the health effects of infectious diseases: are there potential risks for tourists? International Journal of Safety and Security in Tourism and Hospitality 2015; 1(12):1–17.

[7] Ceddia MG, Bardsley NO, Goodwin R, Holloway GJ, Nocella G, Stasi A. A complex system perspective on the emergence and spread of infectious diseases: Integrating economic and ecological aspects. Ecological Economics 2013; 90:124–31.

[8] Crenshaw EM, Ameen AZ, Christenson M. Population Dynamics and Economic Development: Age-Specific Population Growth Rates and Economic Growth in Developing Countries, 1965 to 1990. American Sociological Review 1997; 62(6):974.

[9] Stenholm S, Westerlund H, Head J, Hyde M, Kawachi I, Pentti J, et al. Comorbidity and functional trajectories from midlife to old age: the Health and Retirement Study. J Gerontol A Biol Sci Med Sci 2015; 70(3):332–8. doi: 10.1093/gerona/glu113 25060316 PMC4336333

[10] Phelan JC, Link BG. Fundamental Cause Theory. In: Cockerham WC, editor. Medical Sociology on the Move. Dordrecht: Springer Netherlands; 2013. p. 105–25.

[11] Rudge JW, Hanvoravongchai P, Krumkamp R, Chavez I, Adisasmito W, Chau PN, et al. Health system resource gaps and associated mortality from pandemic influenza across six Asian territories. PLoS One 2012; 7(2):e31800. doi: 10.1371/journal.pone.0031800 22363739 PMC3283680

[12] Farzanegan MR, Gholipour HF, Feizi M, Nunkoo R, Andargoli AE. International Tourism and Outbreak of Coronavirus (COVID-19): A Cross-Country Analysis. Journal of Travel Research 2021; 60(3):687–92.

[13] Farzanegan MR, Feizi M, Gholipour HF. Globalization and the Outbreak of COVID-19: An Empirical Analysis. JRFM 2021; 14(3):105.

[14] Araújo MPD, Nunes VMdA, Costa LdA, Souza TA de, Torres GdV, Nobre TTX. Health conditions of potential risk for severe Covid-19 in institutionalized elderly people. PLoS One 2021; 16(1):e0245432. doi: 10.1371/journal.pone.0245432 33444352 PMC7808625

[15] Ciccone A, Papaioannou E. Human Capital, the Structure of Production, and Growth. The Review of Economics and Statistics 2009; 91(1):66–82.

[16] Testa A, Rennó Santos M, Weiss DB. Incarceration rates and hospital beds per capita: A cross-national study of 36 countries, 1971–2015. Soc Sci Med 2020; 263:113262. doi: 10.1016/j.socscimed.2020.113262 32784099 PMC7398037

[17] Hartvig P, Kjelsberg E. Penrose’s law revisited: the relationship between mental institution beds, prison population and crime rate. Nordic Journal of Psychiatry 2009; 63(1):51–6. doi: 10.1080/08039480802298697 18985517

[18] Verity R, Okell LC, Dorigatti I, Winskill P, Whittaker C, Imai N, et al. Estimates of the severity of coronavirus disease 2019: a model-based analysis. The Lancet Infectious Diseases 2020; 20(6):669–77. doi: 10.1016/S1473-3099(20)30243-7 32240634 PMC7158570

[19] Khalili M, Karamouzian M, Nasiri N, Javadi S, Mirzazadeh A, Sharifi H. Epidemiological characteristics of COVID-19: a systematic review and meta-analysis. Epidemiol Infect 2020; 148:e130. doi: 10.1017/S0950268820001430 32594937 PMC7343974

[20] Historical data (to 14 December 2020) on daily number of COVID-19 cases and deaths by country worldwide [Internet]. European Centre for Disease Prevention and Control (ECDC). [cited 2020 December 14]. Available from: https://www.ecdc.europa.eu/en/publications-data/download-todays-data-geographic-distribution-covid-19-cases-worldwide.

[21] World Development Indicators. [Internet]. World Bank. [cited 2020 April 14] Available from: https://data.worldbank.org/.

[22] Rennie S, Buchbinder M, Juengst E, Brinkley-Rubinstein L, Blue C, Rosen DL. Scraping the Web for Public Health Gains: Ethical Considerations from a ’Big Data’ Research Project on HIV and Incarceration. Public Health Ethics 2020; 13(1):111–21. doi: 10.1093/phe/phaa006 32765647 PMC7392638

[23] DeGuzman PB, Altrui P, Allen M, Deagle CR, Keim-Malpass J. Mapping Geospatial Gaps in Early Identification of Children With Autism Spectrum Disorder. J Pediatr Health Care 2017; 31(6):663–70. doi: 10.1016/j.pedhc.2017.05.003 28688939

[24] Del Valle EPG, García GL, Santamaría LP, Zanin M, Ruiz EM, González AR, editors. 2018 IEEE 31st International Symposium on Computer-Based Medical Systems (CBMS). IEEE; 62018.

[25] Doğaner A. The Role of Wikipedia in providing information on coronavirus to Societies during the COVID-19 Pandemic. Middle Black Sea Journal of Health Science 2020; 6(3):316–24. Available from: URL: https://dergipark.org.tr/en/pub/mbsjohs/issue/58433/781930.

[26] Hox JJ. Multilevel analysis: Techniques and applications. 2. ed. New York: Routledge Taylor & Francis; 2010. (Quantitative methodology series).

[27] Hox JJ, Meij LW. The multilevel regression model. In: Best H, Wolf C, editors. The SAGE handbook of regression analysis and causal inference. London: SAGE Publications; 2014. p. 133–51.

[28] Bradley EH, Elkins BR, Herrin J, Elbel B. Health and social services expenditures: associations with health outcomes. BMJ Qual Saf 2011; 20(10):826–31. doi: 10.1136/bmjqs.2010.048363 21447501

[29] Linden M, Ray D. Aggregation bias-correcting approach to the health–income relationship: Life expectancy and GDP per capita in 148 countries, 1970–2010. Economic Modelling 2017; 61:126–36.

[30] Holz M. Health inequalities in Germany: differences in the ‘Healthy migrant effect’ of European, non-European and internal migrants. Journal of Ethnic and Migration Studies 2021:1–22.

[31] Kennedy S, Kidd MP, McDonald JT, Biddle N. The Healthy Immigrant Effect: Patterns and Evidence from Four Countries. Int. Migration & Integration 2015; 16(2):317–32. Available from: URL: https://idp.springer.com/authorize/casa?redirect_uri=https://link.springer.com/article/10.1007/s12134-014-0340-x&casa_token=mviirvrcyecaaaaa:dniaz00tbwkhdsz39afkmj2m-cvh-fs9wuvfcftyxppdpcm97mpglyb3lqfefislw46ugv5cr_pnb_z4yg.

[32] Commission on a Global Health Risk Framework for the Future; National Academy of Medicine, Secretariat. The Neglected Dimension of Global Security: A Framework to Counter Infectious Disease Crises. Washington (DC): National Academies Press (US); 2016 May 16. doi: 10.17226/21891. 27336117

