# Supplementary material for: Early days of the pandemic—The association of economic and socio-political country characteristics with the development of the COVID-19 death toll
Source: PLoS One. 2021 Aug 31;16(8):e0256736. doi: 10.1371/journal.pone.0256736 (PMC8407552; doi:10.1371/journal.pone.0256736)
Supplement: S1 Appendix — (DOCX) [file pone.0256736.s001.docx]

**S1 Appendix**

Table 2: Worldwide Deaths by COVID-19: Multilevel Analysis of longitudinal country-level data including country fixed effects (not shown)

|  |  |  | Estimates (SE) |  |  |
| --- | --- | --- | --- | --- | --- |
| Model | 1 | 2 | 3 | 4 | 5 |
| (Intercept) | -0.41  (44.11) | -1.63 (42.19) | 0.14  (41.97) | -0.17  (38.04) | -0.74 (43.45) |
|  |  |  |  |  |  |
| **Level I predictors:** |  |  |  |  |  |
| Days(log) | **68.30*****  (10.46) | **67.12***** (10.37) | **68.55***** (9.70) | **69.40***** (7.93) | **69.16***** (7.53) |
| **Cross-Level-Interactions:** |  |  |  |  |  |
| Days(log) * National Wealth | - | -13.38 (7.74) | - | - | -4.94  (6.17) |
| Days(log) * Economic Growth | - | 0.77  (5.79) | - | - | -8.63  (4.90) |
| Days(log) * Hospital beds | - |  | **-10.26***  (5.15**)** | - | **-9.49***  (4.16) |
| Days(log) * Health expenditures | - |  | **-1571.15**** (547.26) | - | -767.16 (526.76) |
| Days(log) * Tourists | - |  | - | **2.09*****  (0.61) | **1.82*****  (0.61) |
| Days(log) * Net migration | - |  | - | **-7.25***** (1.36) | **-6.71***** (1.38) |
| Days(log) * Exposure confirmed infected | **219.41***** (20.79) | **232.66***** (22.44) | **245.13***** (22.83) | **248.59***** (29.58) | **257.28***** (30.66) |
| Days(log) * Age 65+ | 163.74 (233.71) | 308.03 (245.79) | **794.61*** (311.49) | 8.30  (179.14) | **580.19*** (274.71) |
| Days(log) * Age 15-64 | -74.32  (328.94) | 143.83 (352.88) | 7.81  (320.69) | -144.21 (252.06) | 18.08 (274.71) |
| Days(log) * Education rate | 39.65  (65.89) | 93.92 (74.05) | 36.16  (65.20) | 60.46  (49.82) | 40.10 (56.34) |
| Days(log) * Population Size | -0.01  (0.06) | -0.02  (0.06) | -0.03  (0.06) | **-0.15****  (0.05) | **-0.14****  (0.05) |
| Days(log) * Population Density | 3.20  (10.63) | 12.67 (11.84) | -3.97  (65.20) | 2.54  (7.95) | 2.0  (9.08) |
| **Random Effects** |  |  |  |  |  |
| σ^2^_eij_ | 1328 | 1329 | 1329 | 1328 | 1329 |
| σ^2^_u0j_ _intercept_ | 1668 | 1502 | 1484 | 1171 | 1612 |
| σ^2^_u1j_ _slope of days(log)_ | 5977 | 5848 | 5106 | 3274 | 2923 |
| **Fit Measures** |  |  |  |  |  |
| AIC | 24486 | 24476 | 24461 | 24454 | 24424 |
| BIC | 24898 | 24900 | 24884 | 24878 | 24871 |
| LogLik | -12172 | -12165 | -12157 | -12154 | -12135 |
| Observations | 2453 | 2453 | 2453 | 2453 | 2453 |
| n _countries_ | 61 | 61 | 61 | 61 | 61 |

*Remarks:*

Sig codes: p ≤ 0.001***, p ≤ 0.01 **, p ≤ 0.05 *

days(log): -LOG of days before last measurement occasion; Restricted Maximum Likelihood (REML) estimator; all country characteristics were grand mean centered; Fit measures with Maximum Likelihood (ML) estimator: Intra Class Coefficient (ICC) of the unconditional model: 15,4%; software: lme4-package in R
